# Developmental cascades as a framework for primate handedness

**DOI:** 10.3389/fnbeh.2022.1063348

**Published:** 2022-11-07

**Authors:** Eliza L. Nelson

**Affiliations:** Department of Psychology, Florida International University, Miami, FL, United States

**Keywords:** handedness, laterality, infants, primates, developmental cascades

## Introduction

Development matters for understanding handedness. Handedness is a behavioral phenomenon where one hand is used preferentially over the other for manual skills involving one or both hands. Humans are unequivocally right-handed—the ratio of right-handers to left-handers in any population is 9:1 (Papadatou-Pastou et al., 2020). However, this ratio is derived from adults and does not represent hand use across infancy and toddlerhood, which is best characterized by different developmental trajectories (Fagard, 2013; Michel et al., 2014; Nelson et al., 2017; Campbell et al., 2018; Gonzalez et al., 2020). Multiple trajectories occur because handedness is the result of cascading developmental events (Michel et al., 2013). Studying handedness, or any attribute that manifests in an individual, requires an understanding that development is a continuous process of individual-environment interactions that is subject to factors that facilitate, constrain, or alter an individual's progression along developmental paths (Developmental Psychobiology; Michel and Moore, 1995; Michel, 2021, 2022). In this opinion, I argue that handedness is not innate; nor is handedness a culturally entrained trait. Rather, handedness emerges as the result of developmental cascades beginning prenatally. While evidence supports developmental cascades in infant handedness, few studies have examined handedness in nonhuman primates with this lens. To close this gap, I challenge investigators to adopt a developmental cascades framework in primate handedness research.

## Setting the stage: Evidence supports developmental cascades in the emergence of handedness in human infants

**Figure 1:**
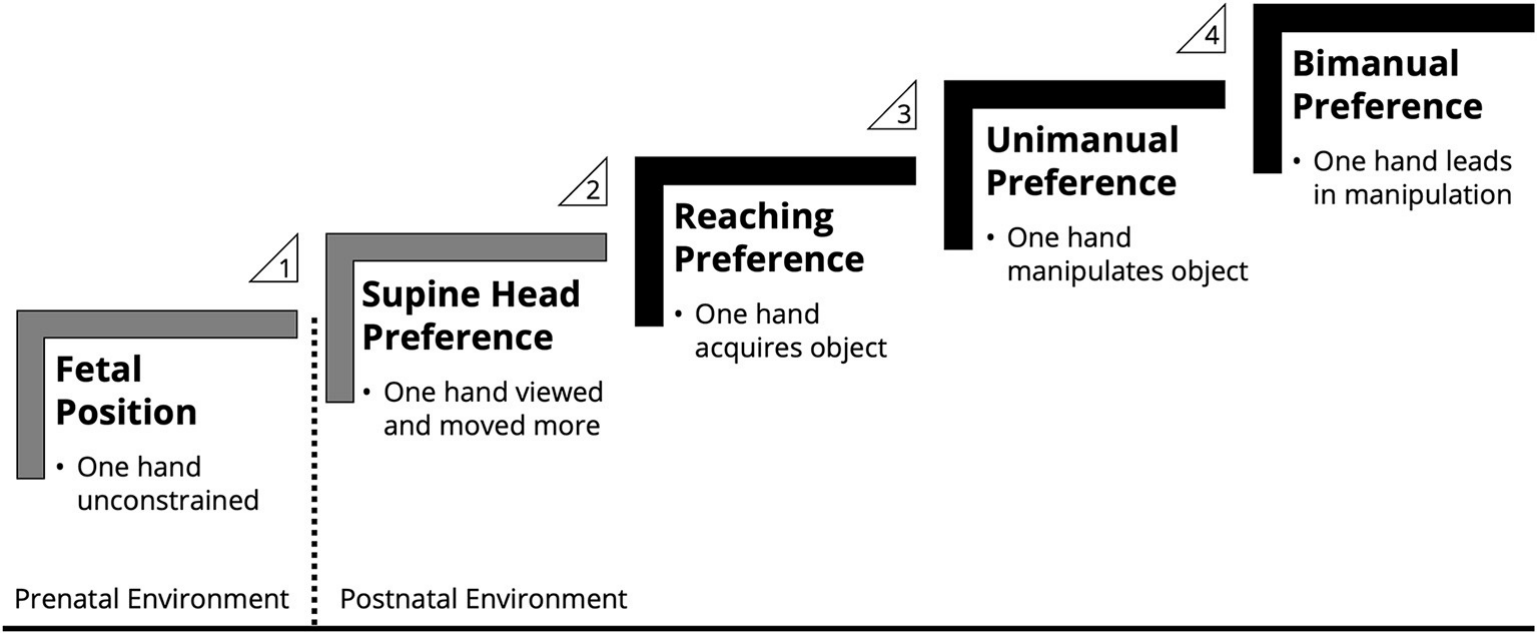
Handedness emerges in human infants *via* cascading developmental events. Data supporting the predictive links in the cascade: (1) fetal position predicts head turn preference: Michel and Goodwin (1979); (2) head turn preference predicts reaching hand preference: Michel (1981); Michel and Harkins (1986); Konishi et al. (1987); (3) reaching hand preference predicts unimanual hand preference: Hinojosa et al. (2003); Campbell et al. (2015); (4) unimanual hand preference predicts bimanual hand preference: Babik and Michel (2016). Gray shading denotes postural asymmetries. Black shading denotes manual asymmetries.

Infant handedness arises from cascading developmental events beginning prenatally (Figure 1; Michel, 2002, 2021; Michel et al., 2013). There is a postural asymmetry in the intrauterine environment as the result of fetal growth— the more the fetus grows, the more restricted its position and movement becomes. The fetus turns head-down in preparation for birth with the head turned such that one ear (typically the right) faces outward. This leftward fetal positioning restricts movement of the left arm, leaving the right arm free to move, and this prenatal asymmetry has been hypothesized to be the basis for human handedness (Previc, 1991). Fetal uterine position, estimated during delivery, predicts postnatal head orientation preference when infants are placed supine (Michel and Goodwin, 1979). Most infants are born in a left position and exhibit a right supine head turn preference. The implication of the head turn preference is that infants receive asymmetric multimodal feedback of their hands. Infants view one hand more than the other, and the hand that is viewed more is moved more (Coryell and Michel, 1978; Michel and Harkins, 1986; van der Meer et al., 1995). Unsurprisingly, which way the infant prefers to turn their head is a strong predictor of which hand they prefer to use for reaching (Michel, 1981; Michel and Harkins, 1986; Konishi et al., 1987). The infant's reaching hand preference in turn predicts their later hand preference for manipulating objects with one hand (for example, shaking, mouthing; Hinojosa et al., 2003; Campbell et al., 2015), which cascades to their hand preference for manipulating objects with two hands wherein the non-preferred hand supports an object for the preferred hand's actions (RDBM, role-differentiated bimanual manipulation; Babik and Michel, 2016). In other words, infants' early environmental experiences are cumulative. Developmental cascades are not constrained to infants with emerging right biases, who are the majority; left biases also follow this pattern from prenatal to postnatal asymmetries as left-handedness emerges. Taken together, data gathered with a Developmental Psychobiology approach to map individual-environment coactions across different behaviors and time have provided sufficient evidence for developmental cascades in the emergence of handedness in human infants. Critically, adopting a cascades framework shifts the emphasis away from attempts to match the adult population-level right bias *via* cross-sectional studies (i.e., examining different ages to determine when the 9:1 right to left ratio is established). Instead, the variability within and between infants can be captured by multiple trajectories within individual behaviors examined longitudinally (e.g., Jacobsohn et al., 2014; Michel et al., 2014). These trajectories, in turn, have predictive value for non-motor developmental outcomes (e.g., Gonzalez et al., 2020). Small changes in one domain can have downstream effects in another domain, and these rich patterns are only detectable when researchers employ a developmental cascades framework (Masten and Cicchetti, 2010; Iverson, 2021).

## Point of view: Scant attention has been paid to testing developmental cascades for handedness in primates

Contrary to human research, infants are poorly represented in the primate handedness literature. Most studies of primate handedness, like research in primate cognition more broadly, have used adult samples (Nelson, 2022; Nelson et al., 2022). Far fewer still are infant primate handedness studies that have measured multiple behaviors at multiple timepoints—prerequisites for testing predictive links in a developmental cascade. Some longitudinal handedness studies in primate infants have only measured a single behavior (e.g., Hook and Rogers, 2000) while others have measured multiple behaviors but at a single timepoint (e.g., Westergaard et al., 1997). Hopkins (2004) hypothesized that early environmental factors may shape the emergence of handedness in primates as they do in humans. Yet only three studies have tested part of the handedness cascade described for human infants (Figure 1) in primates.

Strikingly, evidence from chimpanzees *(Pan troglodytes)* suggests a handedness cascade like humans—neonatal head turn preference predicts later hand preference. Hopkins and Bard (2000) collected two sets of measures of lateral bias in chimpanzee infants. Supine head orientation was observed from sleep over the first 3 months of life during the neonatal period. Juvenile measures collected when chimpanzees were 3–5 years of age included hand preferences for reaching and bimanual manipulation. A right population-level bias was found for supine head turn preference and for bimanual manipulation, although no population-level bias was found for reaching. Subsequently, supine head turn preference significantly predicted bimanual manipulation, but not reaching, hand preference. The lack of a head-hand link for reaching may be due to when reaching data were collected. Reaching is now regarded as a poor measure of hand preference outside of infancy (for a discussion, see Nelson, 2022). Furthermore, the presence of a head-hand link for bimanual manipulation requires further investigation to uncover the potential mechanism. Because head data were collected during sleep, it is unlikely this bias provided asymmetrical feedback of the hands. It is possible that chimpanzee infants do receive asymmetrical manual experience from some other aspect of their early environment, and this possibility should be explored in future work.

An inverse relationship between head orientation and later hand preference has been reported in capuchins *(Cebus apella)*, indicating handedness cascades may differ based on species-typical experiences. Westergaard et al. (1998) recorded the direction of the infant's head while they were riding on their mother's back (i.e., in a prone position) over weeks 1–2 of life. Hand preference was collected from a reaching measure collected at two timepoints when the infant was independent of its mother at weeks 23–24 and weeks 47–48. No population-level bias was found for prone head orientation. Population-level left biases were found for reaching hand preference at both timepoints. Prone head turn preference was negatively correlated to the first reaching preference timepoint, but not the second. These findings confirm that measures of prone head turn preference have not yielded robust patterns of laterality in any infant primate sampled to date, including humans. The reason why a bias is not observed in the prone posture is unknown. Moreover, the longevity of head-hand links is also unknown, which could explain why a relation was found between head turn preference and earlier, but not later, hand preference. Like the chimpanzee infant study, Westergaard et al. (1998) did not record infants' experiences of their hands during head turn data collection. It is possible that the amount of hand viewing or hand movement may moderate the relation between head turn preference and later hand preference, or there could be multiple patterns (i.e., head-hand subgroups). These two possibilities would be masked in simple correlation analyses in a small sample design. Future work in larger samples could address these points.

A study in rhesus macaques *(Macaca mulatta)* was the first to record primate infants' hand activity during head turns by using an experimental, rather than observational, testing procedure, replicating the human finding of greater activity in the hand that is viewed more. Nelson et al. (2011) measured supine head turn preference on days 1, 3, 7, 14, 21, and 30. Hand-to-face contacts were coded from video during head turn trials. Hand preference for reaching to objects was collected between 14 and 44 days of age, and hand preference for bimanual manipulation was collected between 6 and 9 months of age. A population-level left bias was found for head turn and hand-to-face. Monkeys had individual hand preferences for reaching and bimanual manipulation but there were no group-level biases. There was a large effect linking supine head turn preference to hand-to-face activity. However, head turn did not predict either of the later hand preference measures. Nelson et al. (2011) suggested that the discontinuity in the developmental cascade for handedness in rhesus macaques could be the consequence of monkeys not spending time supine under naturalistic conditions (i.e., outside of the experimental head testing procedure) relative to human and chimpanzee infants for whom the supine posture is part of their normal repertoire. In addition, macaques develop at a rate that is approximately four times as fast as their human counterparts (Gunderson and Sackett, 1984). Environmental factors in macaques may have a dampened influence on a handedness cascade due to shortened time to accumulate early lateralized experiences. In addition to a longer period of development, human infants also have immature brains at birth as compared to other primates. These two factors could translate to human infants being more “susceptible to lateralization” from early environmental experiences (Hopkins, 2022). This hypothesis is testable with future research using a developmental cascades framework.

## Bridging the gap: A challenge for primate handedness researchers to adopt a developmental cascades framework

There is vast untapped potential for a developmental cascades framework to shed new light on the evolutionary origins of handedness in primates. Most primate handedness studies have not been developmental (Boulinguez-Ambroise et al., 2022a), failing to capture change over time and any patterns between early asymmetric sensory and motor experiences. While there is some evidence of similarities between human and primate infant cascades, there are also discontinuities that have yet to be explored. Moreover, the work to date has largely been driven by the cascade model for human infants; the links that have been studied may not be applicable to all primates, who have different developmental experiences. To my knowledge, there is no comparable test of the cascade hypothesis in any prosimian species.

A challenge for primate handedness researchers is to identify early candidate behaviors that may contribute the emergence of handedness, and to track these behaviors with rigorous measurement over time in a manner that is species appropriate. Trajectories likely follow different paths for functionally different manual actions like object manipulation vs. communicative gestures (cf. Boulinguez-Ambroise et al., 2022a). Despite the inherent value of developmental cascades data for primate handedness, there are sampling roadblocks to consider such as access to infant primate populations in sufficient numbers for statistical testing, and when infants are available, the ability of the researcher to gather infant-level data. Crowdsourcing data such as efforts by ManyPrimates et al. (2019), and the use of paradigms that permit social group testing may be solutions for these issues.

A developmental cascades framework will benefit from advancements in brain imaging coupled with careful attention to utilizing tasks of comparable complexity across species (Nelson, 2022). MRI research in adult primates of several species has found brain anatomical correlates of hand preference for bimanual manipulation but no such links for reaching hand preference (Hopkins and Cantalupo, 2004; Phillips and Sherwood, 2005; Margiotoudi et al., 2019). Newborn baboons show a leftward asymmetry that increases across development in the planum temporale (Becker et al., 2021, 2022). However, it is unknown if structural brain asymmetries vary with behavioral asymmetries across ontogeny in primates.

A final consideration is that developmental cascades for handedness in primates may be indirectly influenced by temporal social factors such as early mother-infant interactions. Boulinguez-Ambroise et al. (2022b) found that baboon *(Papio anubis)* mother's cradling bias was related to their infant's hand preference for grasping during the first 4 months of life when infants are primarily cradled, and this relation weakened as mothers shifted to carrying the infant dorsally or ventrally and disappeared completely when infants were no longer carried. This effect was driven by right-cradled infants, whereas hand preferences were relatively consistent in left-cradled infants. In a related study, Boulinguez-Ambroise et al. (2020) reported a right shift in cradling in mothers living in high density groups. High social pressure may influence developmental cascades in primates; this hypothesis could be tested by following individuals in groups where the social structure, and thus level of social pressure, changes. Cradling likely influences lateralization for socio-emotional information processing, rather than handedness directly (for a review, see Vauclair, 2022). Adopting a developmental cascades framework will further reveal the role of parental influences and self-generated social and environmental experiences on the emergence of handedness in primates.

## Author contributions

ELN conceptualized and wrote this Opinion.

## Conflict of interest

The author declares that the research was conducted in the absence of any commercial or financial relationships that could be construed as a potential conflict of interest.

## Publisher's note

All claims expressed in this article are solely those of the authors and do not necessarily represent those of their affiliated organizations, or those of the publisher, the editors and the reviewers. Any product that may be evaluated in this article, or claim that may be made by its manufacturer, is not guaranteed or endorsed by the publisher.
